# Density override‐based VMAT optimization to improve robustness at tissue‐air interfaces in head and neck radiotherapy

**DOI:** 10.1002/acm2.70737

**Published:** 2026-09-10

**Authors:** Sagarika Jain, Ashlee Ewing, Michael Weldon, Allison Palmiero, Daniel Christ, Emile Gogineni, Simeng Zhu, Sung Jun Ma, John C Grecula, Darrion L Mitchell, Sujith Baliga, Dukagjin M Blakaj, David J Konieczkowski

**Affiliations:** ^1^ Department of Radiation Oncology James Cancer Hospital The Ohio State University Columbus Ohio USA

**Keywords:** density‐override, HN, VMAT

## Abstract

**Background:**

Volumetric Modulated Arc Therapy (VMAT) is integral to head and neck (HN) radiation therapy; however, plan robustness may be compromised at air‐tissue interfaces. Highly modulated fluence directed at these areas can produce significant unexpected high‐dose regions in the event of minor variations in tissue density.

**Purpose:**

This study characterizes an instability in VMAT planning for HN radiation therapy (RT) occurring at the interface between target volume (e.g. mucosal primary tumor) and internal air spaces (e.g. pharyngeal lumen, sinuses, nasal cavity, etc.). A density‐override based planning technique is presented and validated to mitigate this instability.

**Methods:**

Treatment plans for fifteen HN patients receiving VMAT RT at our institution between 2023 and 2024 were retrospectively analyzed. All patients received 60–69.96 Gy in 30–33 daily fractions using 6 MV photons. To model swelling or tumor growth near the air–tumor interface, verification plans were generated by overriding internal air within 3 mm of the planning target volume (PTV) to soft tissue density (HU = 0). A robustness‐enhancing technique was evaluated, wherein optimization was performed with luminal air overridden to HU = –300, followed by recalculation and renormalization using the original CT HU values. Robustness was then reassessed after overriding luminal air to HU = 0.

**Results:**

Baseline plans demonstrated controlled hotspot magnitudes within the BODY, with a median D0.1cc of 109.0% [Interquartile range: 107.8–110.4]. Simulated tissue‐air interface changes produced substantially elevated hotspots in verification plans, increasing to a median D0.1cc of 115.6% [111.9–126.9], corresponding to a median paired increase of 9.0% (Wilcoxon *p* = 1.22 × 10^−^^4^). Robust plans generated using the proposed density‐override optimization maintained comparable baseline dosimetry (median D0.1cc 108.1% [107.4–108.7]) while demonstrating stable hotspot behavior under simulated density perturbations (median D0.1cc 108.1% [107.1–108.6], *p* = 0.18). Target coverage remained preserved, with robust verification plan D95 values ranging from 99.7% to 100.5% (*p* = 0.26).

**Conclusion:**

A density‐override optimization approach mitigates hotspot formation at air–tumor interfaces, producing VMAT plans that remain dosimetrically stable despite variation at internal air‐tumor interfaces.

## 1 INTRODUCTION

Volumetric Modulated Arc Therapy (VMAT) has become the standard technique for modern Head and Neck (HN) radiotherapy planning.^1^, ^2^ Planning for HN treatments is particularly challenging due to irregular shapes of target volumes and the proximity to multiple organs at risk, which necessitates steeper dose gradients. As a result, HN VMAT plans are often highly complex and heavily modulated to achieve adequate target coverage while respecting organ at risk (OAR) constraints.

Although VMAT offers improved plan quality over 3D techniques, its robustness can be compromised in the presence of heterogeneities such as air cavities within the target volume. These air‐tissue interfaces, common in mucosal primary tumors adjacent to internal air spaces (e.g. pharyngeal lumen, sinuses, nasal cavity, etc.), lack electron equilibrium to allow for full dose build up.^3^, ^4^, ^5^ During optimization, significant fluence may be directed into air cavities to compensate for the absence of dose build‐up and to satisfy overall target coverage objectives. This phenomenon can cause issues if there are even minor interfractional set‐up variations or anatomical changes at the air‐tissue interface, causing the high‐fluence areas to be directed at areas with higher than expected density, resulting in additional scatter and dose buildup, unexpected hotspots,^3^ and potential toxicity.

Previous studies have reported effects of air cavities in target volumes in GYN,^6^ GI,^7^ HN^9^, ^10^ and lung cancers.^11^ Proposed solutions have incorporated modifying the PTV volume to exclude regions of the air cavity.^9^, ^10^, ^11^ However, this approach is not robust to changing air volumes, tumor extension into the air cavity, or daily setup variation.

Recently two studies assessed density overrides for VMAT planning in bronchial regions,^8^ and gastrointestinal gas in the esophagus^7^ respectively. Both revealed that density overrides consistently maintained plan robustness to interfractional anatomical and setup changes. However, this approach has not, to our knowledge, been applied to HN treatment plans with intraluminal air in target volumes.

Here, we report our findings through a retrospective study assessing the dosimetric impact of changing air‐tissue interfaces in HN planning and mitigation strategies using density overrides to improve treatment plan robustness.

## 2 METHODS

### 2.1 Patient selection in retrospective study

We retrospectively identified patients whose target volumes included interfaces between tissue and internal air (e.g. pharyngeal lumen, sinuses, nasal cavity, etc.) where differences in air‐tissue interfaces might occur over the course of the treatment.

Plans for 15 HN patients treated with curative‐intent VMAT radiation therapy (RT) at our institution in 2023–2024 were reviewed following IRB approval. All patients received 60–69.96 Gy in 30–33 daily fractions with 6 MV photon beams. Plans were created in Varian Eclipse v16.1, with Photon Optimizer model PO v15.6 and calculated with AcurosXB (version 16.1).^12^ Dose‐to‐medium is reported with the dose calculation grid set to 0.2 cm. Generally, plans included 3–4 arcs that spanned gantry rotations of 320 degrees per arc, avoiding posterior entry to spare the spinal cord.

An in‐house head and neck RapidPlan model with DVH estimations was employed, with planning priorities focused on achieving 100% of the prescribed dose to 95% of the volume for PTV_High, PTV_Mid, and PTV_Low, corresponding to different dose levels, typically 69.96 Gy, 59.40 Gy and 54.12 Gy respectively. PTV_High_Eval, PTV_Eval_Mid and PTV_Eval_Low structures were created for normalization by cropping the nominal target structures from the external contour by 3 mm internally. Optimization was performed on the full PTV_Eval volumes without separately excluding or accounting for tissue and air components. In all cases, one or more PTVs contained or were adjacent to air cavities.

### 2.2 Density override planning

The modified planning approach used an HU override for internal air cavities within or adjacent to the PTV. The internal air cavity (within or adjacent to PTV) was contoured by segmenting intraluminal air using the 3D brush tool and subsequently intersecting this structure with a 3 mm isotropic expansion of the PTV. The choice of HU override value was guided by two considerations (a) it needed to be high enough to prevent the optimizer from directing excess fluence to achieve nominal dose coverage in air, and (b) low enough to ensure adequate coverage and no cold spots in the PTV in the event of density increase due to soft tissue expansion into this space. A value of −300 HU was selected for clinical implementation based on these considerations and supported by a limited sensitivity analysis discussed below. Plans were optimized via the same optimization objectives as the baseline plan using this modified structure set. After optimization, the HU override was removed before final dose calculation, ensuring the dose is accurately calculated based on the nominal planning CT HU values. The plan was normalized such that 100% of the dose covered 95% of the PTV_High_Eval.

### 2.3 Density override plan robustness analysis

**FIGURE 1:**
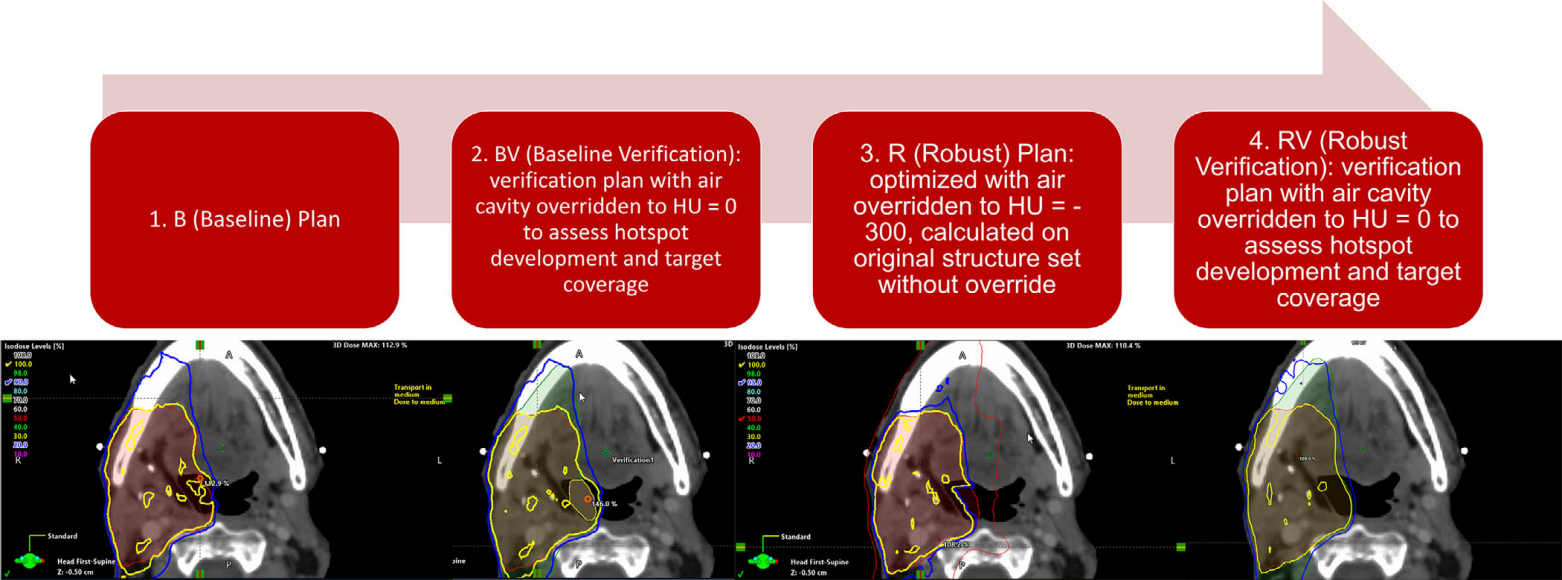
Schematic illustrating the four treatment plan scenarios evaluated in this study using an example patient. (1) Baseline (B): clinically approved treatment plan. (2) Baseline Verification (BV): baseline plan recalculated after overriding intraluminal air cavities within the PTV to HU = 0 to simulate tissue filling and evaluate the dosimetric impact of anatomical change. (3) Robust (R): treatment plan optimized using a density override (HU = −300) applied to intraluminal air cavities within the PTV during optimization only, with final dose calculated on the original patient anatomy. (4) Robust Verification (RV): robust plan recalculated after overriding intraluminal air cavities within the PTV to HU = 0 to evaluate the robustness of target coverage and hotspot development under anatomical change.

The proposed planning approach was validated by evaluating hotspot formation and target coverage when air was replaced by tissue. The workflow is illustrated in Figure 1. Plan 1 (B) served as the baseline plan without any density corrections. Plan 2 (BV) was a verification plan of the baseline, calculated on a duplicated structure set in which internal air within 3 mm of the PTV was overridden to HU = 0, simulating tumor swelling or setup variation at the air‐tissue interface. Plan 3 (R), the robust plan, applied a ‐300 HU override to the air cavity during optimization. Optimization objectives were copied over from the original plan using an in‐house ESAPI script, and no changes were made during optimization. This plan was then copied onto the original structure set with no density overrides for dose calculation. Plan 4 (RV) was a verification plan of Plan 3, calculated on the HU = 0 structure set to assess robustness. Hotspots (D0.1cc), minimum target dose, and Global Dmax were recorded for Plans 2 and 4. Statistical comparisons were performed using paired two‐tailed Student's *t*‐tests and Wilcoxon signed‐rank tests. Cohen's dz was calculated for paired hotspot comparisons. Statistical analyses were performed using MATLAB (version R2025a MathWorks, Natick, MA, USA).

To assess the sensitivity of the planning technique to the choice of HU override value, three representative patient plans were re‐optimized using air density overrides ranging from −1000 HU to −200 HU, following the same optimization approach described above. Hotspots were evaluated by recalculating each plan on a structure set in which internal air within 3 mm of the planning target volume (PTV) was overridden to soft tissue density (HU = 0). The relationship between the override HU value and the resulting hotspot magnitude was then assessed.

## 3 RESULTS

The volume of air cavities ranged from 0.8cc to 26.9cc, accounting from 0.8% to 16.1% of the PTV volume with median 6.3% [IQR: 2.9%–8.8%]. The Global Dmax and D0.1cc within the BODY were evaluated across baseline (B), baseline verification (BV), robust (R), and robust verification (RV) plans in 15 patients. To improve consistency with the skewed hotspot distributions, all hotspot metrics are reported as median [IQR].

Clinically accepted baseline plans demonstrated controlled global Dmax, with a median of 110.5% of prescription dose [109.6%–112.0%]. When the adjacent air cavity was overridden to soft tissue density (HU = 0), baseline verification plans developed substantially larger hotspots, with a median Dmax of 121.6% [115.9%–132%]. This increase was statistically significant by both paired *t*‐test (*p* = 2.7 × 10^−4^, α = 0.05) and Wilcoxon signed‐rank test (*p* = 6.1 × 10^−5^), indicating a systematic increase in hotspot magnitude following tissue‐air perturbation.

**FIGURE 2:**
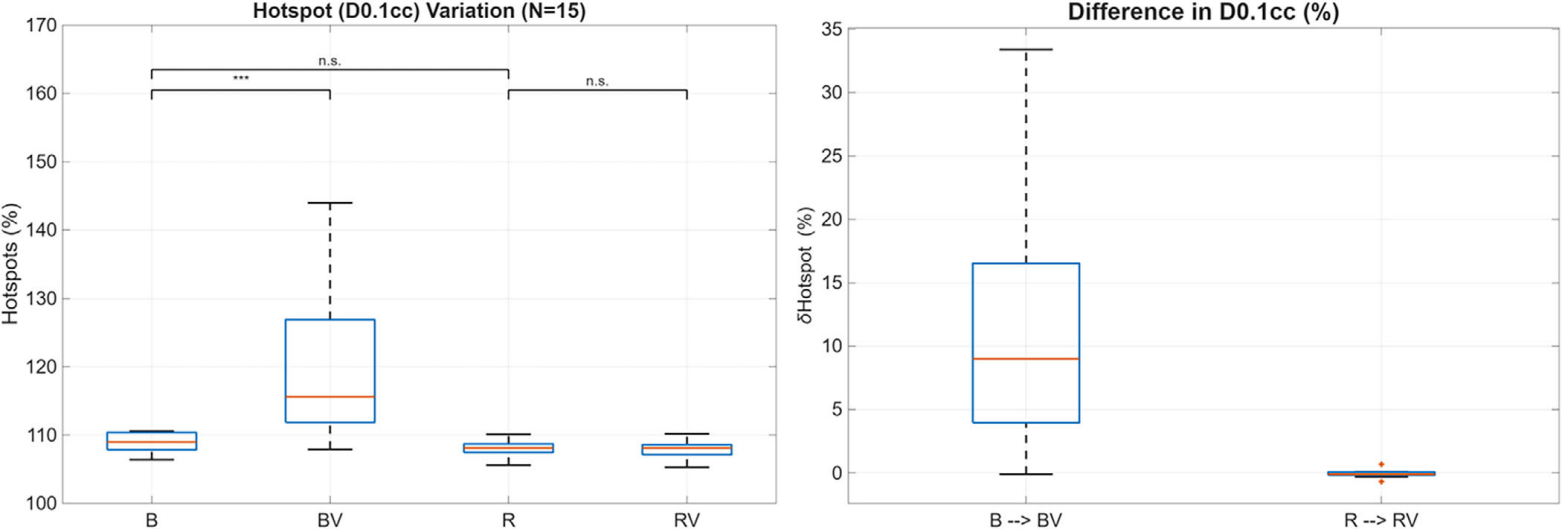
B—Baseline, BV—Baseline Verification, R—Robust plan, RV—Robust plan verification (left) Hotspot ranges across 15 patient treatment plans. BV plans demonstrated a wide range of hotspots. Upon re‐optimizing baseline plans with the robust technique, the resulting hotspots are not significantly different from those of the baseline plans. The robust verification plans also showed no significant hotspot changes despite the introduction of heterogeneities to the calculations. (right) Differences in hotspots that arose in the verification plans relative to their respective plans. Baseline verification plans show up to 34% difference in D0.1cc, while robust verification plans exhibit < 1% difference.

For D0.1cc, baseline plans had a median of 109.0% [107.8–110.4], increasing to 115.6% [111.9–126.9] in BV plans. This corresponded to a median increase of 9.0% (range: 0.1%–33.4%), with a large paired effect size (Cohen's dz = 1.13), further demonstrating sensitivity of hotspot magnitude to tissue‐air interface changes. Wilcoxon signed‐rank testing (*p* = 1.22×10^−^^4^) and paired *t*‐testing (*p* = 6.1 × 10^−4^) confirmed statistically significant increase, as shown in figure 2.

To further assess whether smaller perturbations produced similar effects, three patients with the largest dose perturbations were recalculated using a 2 mm HU = 0 shell. These plans still demonstrated elevated hotspot values of 119.3%, 144.1%, and 155.2% from baseline values of 109.7%, 109.6% and 112.6%, confirming that even modest filling at the tissue‐air boundary can produce clinically relevant hotspot escalation.

**FIGURE 3:**
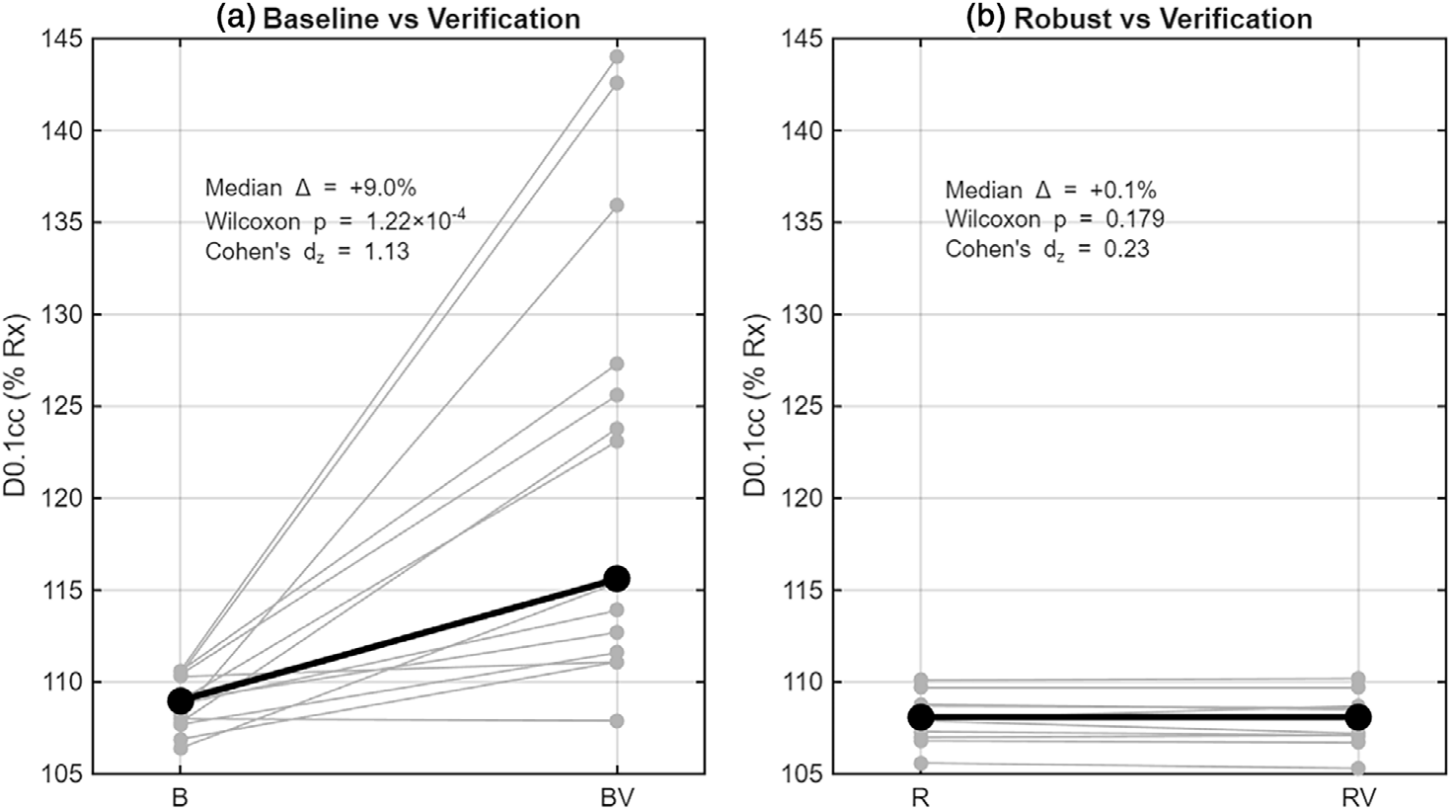
Per Patient Changes in D0.1cc dose from (a) Baseline to Baseline Verification and (b) Robust to Robust Verification plans.

In contrast, robust plans optimized with a ‐300 HU density override (removed for final dose calculation) maintained stable hotspot behavior after HU = 0 perturbation. Global Dmax differences between R→RV ranged from −1.2% to 3.6%, with no statistically significant increase (*t*‐test *p* = 0.54; Wilcoxon *p* = 0.35). D0.1cc values remained nearly unchanged between R (108.1% [107.4–108.7]) and RV (108.1% [107.1–108.6]), with only a small effect size (Cohen's dz = 0.23). The per‐patient changes in D0.1cc are shown in figure 3.

**FIGURE 4:**
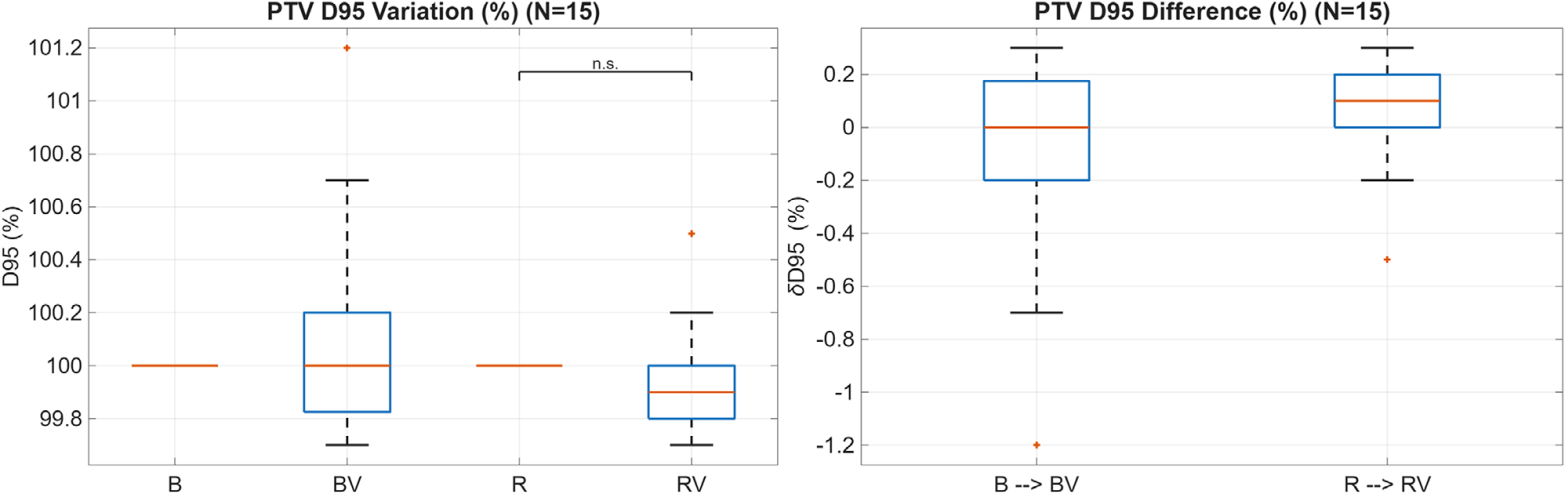
B—Baseline, BV—Baseline Verification, R—Robust plan, RV—Robust plan verification (a) PTV coverage is quantified using D95% between the baseline, baseline verification, robust and robust verification plans. Robust plans were re‐normalized in the TPS, and resulting verification plans led to statistically insignificant changes in coverage. (b) Robust plans did not lose more than 0.5% target coverage dose when tissue‐air changes were simulated in the verification plans.

Both baseline and robust plans were normalized such that 95% of the PTV_Eval_High received 100% of the prescribed dose. The D95(%) values for the robust verification plans ranged from 99.7% to 100.5%, showing no statistically significant change (*p* = 0.26, α = 0.05), as shown in Figure 4.

Furthermore, Total monitor units (MU) were comparable between baseline and robust plans, with median values of 807 [IQR 692.8–853.3] and 780 [689.8 845.5], respectively, with no statistically significant difference (paired *t*‐test *p* = 0.58; Wilcoxon *p* = 0.91), suggesting no substantial increase in delivery burden.

Key OAR dosimetric endpoints remained comparable between baseline and robust plans (Table 1). No statistically significant differences were observed for spinal cord, brainstem, parotid glands, oral cavity, or larynx dose metrics (all Wilcoxon *p* > 0.05), indicating that the proposed optimization strategy improved hotspot robustness without compromising OAR sparing.

**TABLE 1:** Comparison of key organ‐at‐risk (OAR) dosimetric parameters between baseline and robust treatment plans (N = 15). Values are reported as median [interquartile range]. Statistical comparisons were performed using the paired Wilcoxon signed‐rank test.

Dose Parameter (Gy)	Baseline Median [IQR]	Robust Median [IQR]	Wilcoxon *p*‐value
Cord D0.03cc	19.68 [17.64–22.30]	19.84 [17.23–21.38]	0.59
Brainstem D0.03cc	19.50 [18.42–23.41]	19.17 [17.72–21.81]	0.45
Left Parotid Mean	23.14 [21.68–30.43]	23.33 [21.54–30.46]	0.74
Right Parotid Mean	25.60 [22.07–30.82]	26.29 [22.27–31.45]	0.43
Oral Cavity Mean	38.65 [32.56–47.38]	38.67 [32.42–40.48]	0.93
Larynx Mean	51.99 [34.04–62.73]	50.68 [35.06–62.66]	0.98

**FIGURE 5:**
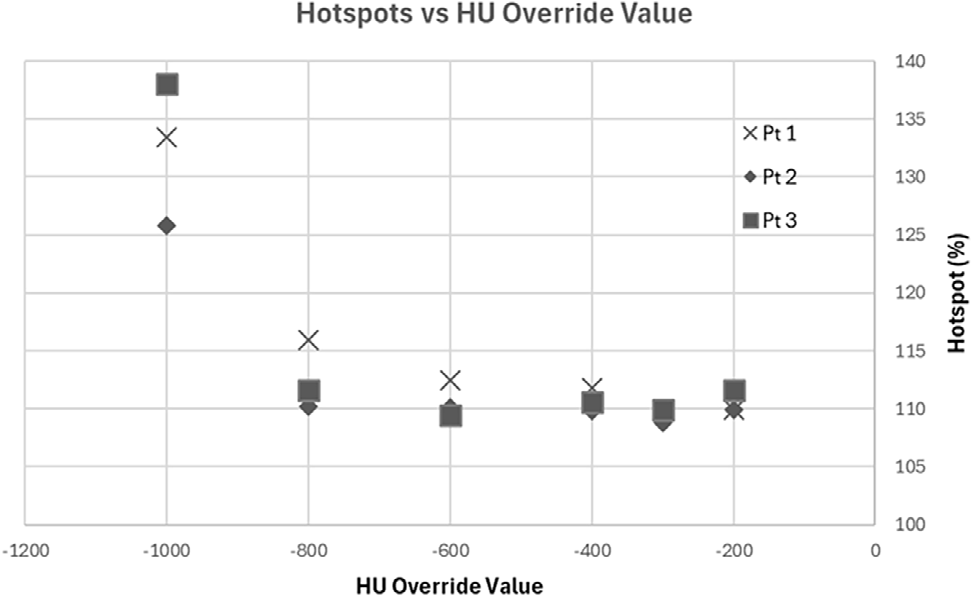
Global Dmax developed in baseline verification plans when the same plan was optimized using different HU override values. Dmax decreases significantly between ‐1000 HU and ‐800 HU, and then stabilize across to < 113% at ‐600 HU and higher.

A limited sensitivity analysis was performed in three selected high‐perturbation cases to evaluate the impact of varying HU override values. Hotspot magnitude decreased substantially as the override increased from −1000 HU to −800 HU and stabilized between −600 HU and −200 HU across all three cases (Figure 5). Within this plateau region, hotspot magnitude showed minimal dependence on the specific override value, supporting the selection of −300 HU as a practical mid‐range value for clinical implementation.

## 4 DISCUSSION

This paper reports our institutional experience with adopting a density‐override based treatment planning technique to improve the robustness of head and neck (HN) plans involving air cavities, aiming to minimize the development of unexpected hotspots while still maintaining target coverage. This robustness is achieved by applying a density override to the air cavity region during the optimization process but removing it for the dose calculation, thereby preventing excess fluence deposition within the cavity during the optimization. This process adds a few minutes to overall planning time, but substantially improves robustness, potentially reducing both mucosal toxicity and the need for replanning due to interfractional changes.

Although this study was limited to the head‐and‐neck region, similar internal density fluctuations are encountered in GI, GU, and GYN treatments (e.g., rectal or bowel gas), suggesting that the general concept of density override may have broader applicability. However, the optimal override value is likely site‐ and anatomy‐specific. In the head‐and‐neck region, internal air cavities are often anatomically constrained and may remain in relatively consistent proximity to high‐dose target volumes, making a partial low‐density override a practical robustness strategy. In contrast, transient bowel gas in the abdomen or pelvis may exhibit greater day‐to‐day positional variability, where alternative override approaches, such as water‐equivalent density, may be more appropriate.

Density overrides have been used in lung planning to improve dose calculation accuracy in heterogeneous media,^13^ and in gastrointestinal^7^ radiotherapy to mitigate target under‐ or over‐coverage caused by variable intestinal gas pockets. In contrast, the objective of the present work represents a novel application of this principle: the density override is used to improve plan robustness against hotspot formation arising from optimizer fluence instability at air–tissue interfaces during VMAT optimization in head and neck tumors. Compared with previously reported bronchial or gastrointestinal cases, highly modulated head‐and‐neck VMAT plans present greater complexity, steeper dose gradients, and tighter target–OAR tradeoffs, increasing sensitivity to localized density perturbations at air–tissue interfaces.

Other studies^6^, ^11^ have looked at excluding air cavities from the target when optimizing to avoid hotspots in tissue‐air interfaces, but this approach may not necessarily ensure robustness in case of anatomical changes. This approach risks target undercoverage if transient air pockets resolve or anatomy changes during treatment. Adachi et al^8^ employed a similar technique of CT‐value override to peri‐bronchial tumors and assessed hotspots and heterogeneity index changes in case of interfractional motion. However, the technique employed in our study is different since we propose re‐optimizing on an overridden structure set but calculating dose on the original structure set. This distinction is clinically relevant because it seeks to improve perturbation robustness without altering the final physical dose calculation environment used for treatment evaluation and delivery. Furthermore, we demonstrate the effectiveness and robustness of this technique using clinically delivered plans with substantial modulation for organ‐at‐risk (OAR) sparing in head‐and‐neck cancers. Such highly modulated plans may exacerbate the issue of fluence deposition into low‐density regions, an effect that may not be adequately captured in studies relying solely on simplified or virtual phantoms.

Other mitigation strategies for interfractional air–tissue changes include fluence smoothing and robust photon optimization. However, fluence smoothing in Eclipse VMAT is largely planner‐dependent and not explicitly designed to account for density uncertainty, limiting reproducibility across patients. While it may reduce modulation, it does not directly address the underlying air–tissue perturbation and may compromise conformity or OAR sparing. Robust photon optimization may offer a more systematic solution by explicitly incorporating uncertainty, but its availability remains limited across treatment planning systems and clinical workflows. Formal comparative evaluation of these strategies would be valuable for future investigation.

This study has several considerations. First, while a value of −300 HU was selected for clinical implementation, sensitivity analysis showed that hotspot mitigation was achieved across a broad range of HU override values. This suggests that the effectiveness of the technique is not highly sensitive to the specific override value chosen; however, the optimal selection of HU override values under varying anatomical conditions warrants further investigation. Second, although the 3 mm HU override represents a simplified and binary perturbation, it was intentionally selected to demonstrate that even minor density variations immediately adjacent to the PTV can result in substantial hotspot formation. This approach isolates sensitivity to local HU changes rather than modeling the full spectrum of anatomical variability. Finally, the sample size is limited, although despite this fact the phenomenon was consistent and highly statistically significant. Finally, this retrospective study design was not able to assess correlation between plan hotspots and patient toxicity, which is essential for assessing the clinical relevance of the observed dosimetric differences. Future studies including larger patient cohorts and evaluation of mucosal toxicity outcomes would help clarify the potential clinical impact of the dosimetric improvements observed with this technique.

## 5 CONCLUSION

In cases requiring full dose to the interface between target volume and luminal air, standard VMAT plans can exhibit unstable behavior due to insufficient region for dose buildup, resulting in clinically unacceptable hotspots with even minor variations in target geometry—variations that may not conventionally trigger re‐planning. We present and validate a density‐override based HN VMAT treatment planning technique for cases involving internal air‐tissue interfaces. This technique preserves baseline plan quality and target coverage but demonstrates marked reduction in hotspot formation in the event of interfactional anatomical or setup variation at the air‐tissue interface.

## AUTHOR CONTRIBUTIONS

**Sagarika Jain**: Conceptualization; data curation; formal analysis; investigation; visualization; writing—original draft preparation. **Ashlee Ewing**: Conceptualization; data curation; investigation; validation. **Michael Weldon**: Conceptualization; writing—review and editing. **Allison Palmiero**: Writing—review and editing. **Daniel Christ**: Conceptualization; methodology; writing—review and editing. **Emil Gogineni**: Writing—review and editing; resources. **Simeng Zhu**: Writing—review and editing; resources. **Sung Jun Ma**: Writing—review and editing; resources. **John Grecula**: Writing—review and editing; resources. **Darrion Mitchell**: Writing—review and editing; resources. **Sujith Baliga**: Writing—review and editing; resources. **Dukagjin Blakaj**: Writing—review and editing; resources. **David Konieczkowski**: Conceptualization; project administration; supervision.

## CONFLICT OF INTEREST STATEMENT

The authors declare no conflicts of interest.

## ETHICAL STATEMENT

This study was approved by the Institutional Review Board at The Ohio State University (Study ID: 2023C0067).

## Data Availability

The data supporting the findings of this study are available from the corresponding author upon reasonable request.
